# The sense of coherence and styles of success in the medical career: a longitudinal study

**DOI:** 10.1186/s12909-014-0254-5

**Published:** 2014-11-28

**Authors:** Malgorzata Tartas, Maciej Walkiewicz, Waldemar Budzinski, Mikolaj Majkowicz, Krzysztof Wojcikiewicz

**Affiliations:** Department of Psychology, Medical University of Gdansk, Tuwima 15 Street, 80-210 Gdansk, Poland; Polish Chamber of Physicians in Gdansk, Sniadeckich 33 Street, 80-204 Gdansk, Poland

**Keywords:** Sense of coherence, Longitudinal study, Job satisfaction, Medical students, Medical staff, Professional success

## Abstract

**Background:**

Sense of coherence is related to well-being, stress and life satisfaction among medical students and physicians. The purpose of the study was to investigate relation between sense of coherence during medical education and styles of success in the medical career.

**Methods:**

The participants were first examined when they applied to medical school in 1999. Questionnaires were given to these students each academic year from 2000 to 2005. Also, 54 medical doctors who had participated in the first phase of the study completed a questionnaire in 2009, four years after graduation. The baseline questionnaire measured the sense of coherence. The follow-up questionnaire included measures of quality of life, work stress and burnout, satisfaction with medicine as a career, and professional competency.

**Results:**

Medical students with the highest sense of coherence later have the highest quality of life and income, and are the least overwhelmed by work stress, but they also show the least satisfaction with medicine as a career and a low level of professional competence.

**Conclusions:**

Antonovsky’s SOC-29 questionnaire can be used to identify specific tendencies in the development of the medical career. Our results may be useful to medical school admissions officers and resident selection committees, in order to identify candidates at risk for failure.

## Background

### Models of success in a medical career

Research has provided evidence that there is no universal model describing success in a medical career, including quality of life. Current information about the quality of life of Polish physicians can be found in a report entitled “Social Diagnosis: Objective and Subjective Quality of Life in Poland”. The ‘Social Diagnosis’ project is probably the world’s largest longitudinal psychological study of quality of life in the population of a specific country [1-6]. The Social Diagnosis is based on panel research; authors return to the same households every few years, with the first sample being taken in the year 2000 (n = 26,178; the population of Poland is approximately 38,204,000). The follow up took place three years later, and since has been repeated every two years. The survey is always conducted in March to aid the elimination of the seasonal effect. Since 2009 measurements ran into the first half of April due to the size of the sample. The results of the Social Diagnosis reveal not only the current state of Polish society, but allow us to follow how it has changed over the last ten years. The results of this study also characterize well-being and life satisfaction in Polish medical doctors in comparison to other occupational groups. This study was our inspiration to develop a model of success in the medical career. The background of presented specific model was based on well-being and life satisfaction, along with several complementary factors mentioned by Gattiker and Larwood in their model of career success, such as job success (performance, happiness at work) and financial success [7].

After a review of the literature, we decided to include work stress and burnout as symptoms of difficulty and failure in response to job expectations, and thus important negative measures of success in the medical career [8-12]. In a previous study we determined which psychological factors predict success in a medical career [13]. The methodology and the sample were exactly the same as in the present study. We established a model based on the model of success existing in the literature [7] and a Polish longitudinal psychological study of quality of life [1-6]. The independent variables that were measured function as predictors of success in presented model were: academic achievement (high school final exam results, medical school admissions test scores, and grade point average in each of the six years of medical school); the sense of coherence; depression; anxiety; coping strategies; value system; the need for social approval. While the markers of this success were: postgraduate medical competence; satisfaction with medicine as a career; work stress and vulnerability to burnout; quality of life [13].

We found that academic achievement explains only professional competence. Satisfaction with medicine as a career, the level of work stress and burnout related to performing this job, and the quality of life of these physicians are conditioned by psychological characteristics. One asset of this model of factors related to success in a medical career would seem to be the fact that most of the differentiated indicators are dependent also on psychological characteristics, such as sense of coherence. Therefore, success in the medical career seems to be the consequence of the level of personality structure integration, and not a simple result of the medical education process. In the context of the data our goal was to find out areas that might prove useful to predict professional development in the medical career.

In another previous study, we used cluster analysis to identify styles of success in the medical career [14]. We identified three styles, based on significant differences between three clusters in terms of postgraduate medical competence, satisfaction with medicine as a career, work stress and burnout, and quality of life.

**Figure 1:**
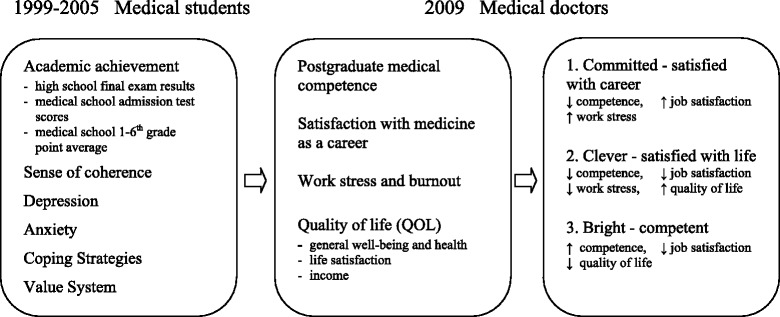
**Model of success predictors and markers of success four years after graduation.**

The physicians who belonged to the first cluster had the lowest medical competence, but they declared the highest level of satisfaction with medicine as a career. At the same time, they had the highest level of work stress and burnout. They were not very happy and satisfied with life, even though they obtained a high income. We may assume that these physicians were the most committed to their work. We have termed this first style “Committed - satisfied with career”. Physicians who fit the second style had low professional competence. They declared the lowest level of satisfaction with medicine as a career and the lowest level of work stress and burnout. On the other hand, their quality of life was the highest. They were the happiest, the most satisfied with life, and they obtained the highest income. It would be reasonable to suppose that these physicians were the least committed to their work, but they derived the most benefit from it. This second style we called “Clever - satisfied with life”. Physicians belonging to the third cluster were the most competent, but they were dissatisfied with medicine as a career. They declared a moderate level of work stress and burnout. Their quality of life was the lowest. They were the saddest and the most dissatisfied with life, and their income was the lowest. This was the group of doctors who were the most competent, but they had problems with managing their lives. This third style was called “Bright - competent” (see Figure 1) (Table 1).

**Table 1:** **Descriptions of the three styles of success in a medical career dimensions: cluster analysis (Bineary Euclidean distance measure and Ward’s linkage method)**

	**1. Committed satisfied with career**	**2. Clever satisfied with life**	**3. Bright unsatisfied**			**1 vs 2**	**1 vs 3**	**2 vs 3**
	**n = 14**	**n = 20**	**n = 16**					
	**M ± SD**	**M ± SD**	**M ± SD**	**F (2;47)**	**P**	**Tukey’s**	**post-**	**hoc test**
Postgraduate medical competence	−0.62 ± 0.61	−0.50 ± 0.53	1.17 ± 0.61	48,256***	<0.001	0,53	<0.001***	0,00**
Satisfaction with medicine as a career	0.95 ± 0.68	−0.47 ± 0.47	−0.24 ± 0.85	20,180***	<0.001	<0.001***	<0.001***	0,31
Work stress and burnout	0.33 ± 0.48	−0.27 ± 0.43	0.07 ± 0.45	7,365***	<0.001	<0.001***	0,13	0,03*
QOL General well-being	−0.29 ± 0.81	0.49 ± 0.55	−0.35 ± 0.60	9,499***	<0.001	<0.001***	0,80	<0.001***
QOL Life satisfaction	−0.60 ± 0.78	0.76 ± 0.45	−0.89 ± 0.86	29,090***	<0.001	<0.001***	0,26	<0.001***
QOL Income	3.57 ± 0.51	3.80 ± 0.41	2.63 ± 0.89	16,803***	<0.001	0,31	<0.001***	<0.001***

*p < 0.05; **p < 0.01; ***p < 0.001.

Sense of coherence in the range of factors mentioned in the literature and in our previous findings appears as significant for predicting success in the medical career. Taking this under consideration it seems to be valuable to analyze it’s linking to styles of success.

### Sense of coherence

In the 1970s, an Israeli sociologist of medicine, Aaron Antonovsky, developed the concept of what he called “salutogenesis,” along with a research methodology to study its ramifications [15,16]. Rather than focusing on the mechanisms underlying illness (pathogenesis), he tried to identify the origin of health (salutogenesis). His model was based on the premise that stress and difficulties, being integral elements of human existence, cause every individual to develop characteristic ways of coping with them. The basic concept of Antonovsky’s model was the sense of coherence, defined as a global orientation that expresses the extent to which one has a pervasive, enduring, but dynamic feeling of confidence that:

- the stimuli emerging from one’s internal and external environments in the course of life are structured, predictable, and explicable (comprehensibility);
- the resources are available to meet the demands posed by these stimuli (manageability);
- these demands are challenges that are worth the investment and engagement they require (meaningfulness).

According to Antonovsky, a strong sense of coherence leads to improved health, but the dichotomy of wellness and illness is the wrong point of departure. Instead, wellness and illness should be viewed as a continuum [16]. As long as we possess the slightest spark of life, we also possess, in some sense, a degree of health. The salutogenetic perspective means that we examine the place of each individual on this continuum at a specific time. At the same time, the concept of the continuum makes it possible to view all people from a pathogenic perspective as partly ill, objects of constant exposure to biopsychosocial risk factors [17]. Salutogenesis focuses on health, rather than pathology. Many studies have attempted to relate the sense of coherence to health variables, such as psychological well-being and mental health [18-22] stress and adaptive coping strategies [23-25] and social support [26,27]. People with a low sense of coherence also cope inefficiently with work-related environmental strain [28] and have a tendency to burnout [29,30]. The sense of coherence is also related to grades at university [31].

Upon analyzing the existing scientific reports on the sense of coherence in the medical profession, we observed that such studies are few in number, and relate only to certain aspects of the problem. A sense of coherence correlates with health behaviors, optimism and self-efficacy in healthy college students [32] and shows significant positive correlation with mental health and negative correlation with psychological distress among medical students [33,34]. Students with a high sense of coherence are more able than others to deal with problems associated with university life, and students who were able to deal with their problems successfully were more likely than others to have high levels of achievement [35]. What is more, the lack of a sense of coherence is an important risk factor for chronic work-related stress in young physicians [36] and has proven to be strongly-protective against work stress [37]. A sense of coherence also proved to be a protective factor for health and life satisfaction in physicians [38] and to interact with job-related emotional strain [39]. This factor is connected with the choice of medical specialization. Surgical or anesthesiological residents show the highest scores for the sense of coherence, while psychiatry residents have low scores [40].

### The goal of the study

The previous research cope with significance of sense of coherence in specific aspects of medical education and career. These interesting data does not give an overall view of the function of this global orientation expressing the level of feeling of confidence while meeting the demands and resources. The presented study was designed to describe role of the sense of coherence during medical education and to clarify how it determines styles of success within four years after graduation.

## Methods

### Institution

The Medical University of Gdansk is the largest medical school in northern Poland and educates (in Polish and English) more than 5000 undergraduate and postgraduate students in four faculties: Health Sciences, Medicine, Pharmacy and Biotechnology. The admission requirements are based on the high school final examination results, and until 2005 the medical school admissions test. The latter test included three basic subjects: biology, chemistry and physics. It was used locally only and prepared by the Medical University of Gdansk. The university accepted those candidates who achieved the best test results; in 1999, it was the best 320 people for the first year of study. Medical training in Poland lasts for six years, and is divided into two years of basic science and four years of clinical training. Graduates must complete one year of internship, providing medical assistance under supervision, mainly in hospital settings. They gain a license to practice medicine when they pass the State Examination for Medical Doctors. The test results determine whether or not further medical specialization will be possible.

### Participants

The first part of the study took place a few days before the admission test in June 1999. All individuals (n = 940) who had applied to the Medical University of Gdansk received a letter with questionnaires (the response rate was 39%, n = 365). Only those who passed the admission exam were taken into consideration for the purposes of our research (n = 320). The procedure was repeated subsequently at the end of every academic year (2000–2005). Questionnaires were sent to all medical students.

Over a period of four years after graduation, the authors cooperated with institutions responsible for postgraduate medical education in Poland. The Medical Examination Centre in Poland provided access to the examination results of postgraduates (the State Examination for Medical Doctors) for 268 identified physicians (n = 268 of 320, an 84% response rate). We also cooperated with the Polish Chamber of Physicians in Gdansk, Poland, where we found the addresses of 255 medical doctors who had participated in the first part of the study. The response rate in this group was 21% (n = 54). The mean age of respondents as of 1 July 2009 was 29.5 ± 0.8 years (69% female). The response rates are shown in Table 2.

**Table 2:** **The response rates**

	**General response rate**	**Final sample response rate**
Admission	n = 178 of 320 (56%)	n = 31 of 54 (57%)
First year	n = 178 of 320 (56%)	n = 32 of 54 (59%)
Second year	n = 129 of 280 (46%)	n = 22 of 54 (41%)
Third year	n = 127 of 302 (42%)	n = 23 of 54 (43%)
Fourth year	n = 121 of 288 (42%)	n = 21 of 54 (39%)
Fifth year	n = 58 of 271 (21%)	n = 10 of 54 (19%)
Sixth year	n = 138 of 240 (57%)	n = 19 of 54 (35%)

This longitudinal research programme was conducted in accordance with the guidelines of the Bioethics Committee of the Medical University of Gdansk, Poland, which reviewed and approved the project.

### Measures

The sense of coherence at admission and during medical school was measured by SOC-29 questionaire. The SOC-29 is based on the concept of salutogenesis by Antonovsky. He introduced his concept to describe whether or to which extent a person finds his or her environment and life circumstances understandable, manageable, and meaningfull. It is a self-rated instrument and has 29 items (each item scores from 1 - which means - ”very often” to 7 - which means “rare or never”, total score ranging from 29 to 203). High scores should be associated with higher levels of SOC [16,41,42].

The assessment of success in the medical career included postgraduate medical competence, work stress and burnout, satisfaction with medicine as a career, and quality of life.

The first parameter of success in medical career - medical competence - was measured by examination results on the State Examination for Medical Doctors, supplied by the Medical Examination Centre in Poland. This exam is administered during the postgraduate internship, and it is required to gain a license to practice medicine. The results determine whether or not further medical specialization will be possible. The exam is organized by the Medical Examination Centre every spring and autumn. The exam starts at the same moment in eleven districts in Poland. It is a multiple choice test. The subject matter includes: internal medicine, paediatrics, surgery, gynaecology and obstetrics, psychiatry, family medicine, emergency medicine and intensive care, oncology, bioethics and medical law, public health, medical jurisdiction.

The second parameter of success in a medical career - burnout - was measured by the Maslach Burnout Inventory (MBI), which has three sub-scales: Emotional Exhaustion, Depersonalisation, and Personal Accomplishment [43,44].

The third parameter of success in a medical career - satisfaction with medicine as a career - was measured by a self-designed survey based on the Cantril’s Scale method, where 1 means “very low” and 10 means “very high” (Cronbach’s alpha = 0.80; r = 0.67)^a^.

The fourth parameter of success in a medical career - quality of life (QOL) - was measured by a questionnaire derived from “Social Diagnosis: Objective and Subjective Quality of Life in Poland” [1-6].

Quality of life consisted of:

- General well-being and health.

It consists of two different questions (Cronbach’s alpha = 0.74, r = 0.40):

- Taking under the consideration your life during last two weeks, could you say it was: unhappy; not happy; quite happy; very happy.
- Taking under the consideration your whole life, could you say it was: awful; unhappy; not very successful; neither good nor bad; pretty good; successful; great).

- Life satisfaction - 22 questions about different aspects of human life: social, financial, surroundings and health (Cronbach’s alpha = 0.83; r = 0.25);

The question was - Please assess the individual aspects of your life, and say how much are you satisfied of them: 1 - very satisfied; 2 - satisfied; 3 - quite happy; 4 - quite dissatisfied; 5 - dissatisfied; 6 - very dissatisfied; 0 - not applicable.

Children; possibility to satisfy one‘s nutritional needs; marriage; own educational level; own health condition; future prospects; relationships with close family members; sexual life; relations with colleagues and superiors; safety in the place of residence; relationships with friends (group of friends); place of residence; own life achievements; the level of available goods and services; housing conditions; manner of spending leisure time; the financial situation of the family; current income of the family; work; moral standards in one‘s environment; the situation in the country.

- Size of income.

### Statistical analysis

All the statistical methods were used because of the methodology of exploratory research where the use of the subsequent analyses is dependent on the previous outcomes.

In the earlier phase of our research, cluster analysis was used (the Binary Euclidean distance measure and Ward’s linkage method) to identify styles of success in the medical career. Cluster analysis uses mathematical algorithms to find groups (styles, clusters, types) of homogeneous items or persons that are similar to each other and that differ from other groups. In the present study, then, ANOVA analysis of variance was used to determine the differences between clusters during medical studies. The number of groups differentiated on the basis of the cluster analysis was not distinct. The three clusters differentiated initially were the result of a comparative analysis of several models. The F statistic and effect size were the justification for the choice of a three group model as an explanation of most of the variances. It appeared that the sense of coherence variable, for instance, differentiated specific styles of success even during medical study, and seems to be a relatively stable characteristic. This would imply that the results of ANOVA show differences due to the family wise data error. In order to reduce this error in the third step, we used discriminant analysis (with the backward method using Ward’s estimate). The predictors were variables from ANOVA, and the dependent variables were clusters (styles of success).

The survey research was conducted according to the longitudinal paradigm for 10 years. The results of such studies often lack a significant amount of random missing data. To remove the data gaps, they were replaced by linear interpolation using existing past data as predictors in the same or similar variables.

## Results

We explored the relation between the styles of success in a medical career we identified in our previous research [14] and the dynamics of the sense of coherence at admission and during the medical career. We found significant differences in the level of a general sense of coherence among the groups defined by the styles of success during medical school, from admission to the fifth year. The “Clever” students had the highest level of general sense of coherence during medical school. They had a higher level than other styles at baseline, and in the first, second (only higher than the “Bright”), fourth and fifth years of medical school (Mean 0 = 145.00, Mean 1 = 149.80, Mean 2 = 145.40, Mean 4 = 142.50, Mean 5 = 146.60).

There are significant differences in the level of the first parameter of the sense of coherence, i.e. comprehensibility, throughout medical school, though not at admission. The “Clever” students have the highest level of comprehensibility. This level is higher than that of the other styles from the first to the sixth year of medical school (Mean 1 = 52.90, Mean 2 = 51.40, Mean 3 = 50.20, Mean 4 = 49.60, Mean 5 = 51.00, Mean 6 = 51.10).

We found significant differences in the second parameter of sense of coherence, i.e. manageability, among the groups defined by styles of success at admission and during the first, fourth and fifth years. The “Clever” students have the highest level of manageability. They have a higher level than the other styles in the baseline, first, fourth and fifth years of medical school, and a higher level than the “Bright” students during the first year (Mean 0 = 53.30, Mean 1 = 53.90, Mean 4 = 53.20, Mean 5 = 53.80).

We found significant differences in the third parameter of the sense of coherence, i.e. meaningfulness, at admission and during the baselina, first, second and fifth years. The “Clever” students had a higher level of meaningfulness than did the “Bright” year (Mean 0 = 43.10, Mean 1 = 43.00, Mean 2 = 41.10, Mean 5 = 41.80).

We did not find any significantly differences in “sense of coherence” between the “Committed” students and the “Bright” students (see Table 3).

**Table 3:** **Means (± standard deviation) for groups representing the three styles of success in a medical career, in terms of the sense of coherence (SOC-29), at admission and during medical school**

	**1. Committed - satisfied with career**	**2. Clever - satisfied with life**	**3. Bright-competent**				**1vs2**	**1vs3**	**2vs3**
	**n = 14**	**n = 20**	**n = 16**						
	**M ± SD**	**M ± SD**	**M ± SD**	**F (2;47)**	**P**	**Eta** ^**2**^	**Tukey’s**	**post- hoc**	**test**
**General sense of coherence**									
Year 0	125.00 ± 25.50	145.00 ± 21.94	125.63 ± 20.69	4.543	.016*	.19	.04*	.99	.04*
Year 1	126.14 ± 23.80	149.80 ± 23.46	116.50 ± 20.57	10.353	.001***	.44	.01*	.48	.00**
Year 2	127.57 ± 21.15	145.40 ± 22.48	122.63 ± 21.95	5.406	.008**	.23	.06	.81	.01*
Year 3	124.14 ± 22.56	140.80 ± 26.16	122.25 ± 18.22	3.603	.035*	.15	.10	.97	.05
Year 4	122.43 ± 25.68	142.50 ± 24.41	123.63 ± 15.41	4.552	.016*	.19	.03*	.99	.04*
Year 5	122.43 ± 16.52	146.60 ± 24.46	123.25 ± 15.31	8.625	.001**	.37	.00**	.99	.00**
Year 6	129.43 ± 24.66	143.20 ± 29.49	125.00 ± 17.99	2.624	.083				
**Comprehensibility**									
Year 0	43.14 ± 9.80	48.60 ± 13.37	48.25 ± 9.98	1.335	.273				
Year 1	41.14 ± 11.41	52.90 ± 8.96	41.00 ± 4.90	11.033	.001***	.47	.00**	.99	.00**
Year 2	39.86 ± 10.34	51.40 ± 6.75	43.75 ± 6.88	9.419	.001***	.40	.00**	.38	.02*
Year 3	41.14 ± 9.91	50.20 ± 9.15	43.50 ± 6.11	5.288	.008**	.23	.01*	.73	.06
Year 4	40.14 ± 11.16	49.60 ± 7.71	44.63 ± 5.29	5.561	.007**	.24	.01*	.30	.18
Year 5	38.43 ± 7.62	51.00 ± 9.58	43.88 ± 4.21	11.412	.001***	.49	.00**	.14	.02*
Year 6	40.71 ± 10.04	51.10 ± 9.44	44.00 ± 4.41	6.950	.002**	.30	.00**	.54	.04*
**Manageability**									
Year 0	45.00 ± 8.02	53.30 ± 9.38	46.38 ± 7.21	5.011	.011*	.21	.02*	.90	.04*
Year 1	46.86 ± 7.89	53.90 ± 10.92	42.25 ± 8.27	7.111	.002***	.30	.09	.38	.00**
Year 2	49.00 ± 9.20	52.90 ± 11.30	45.50 ± 6.04	2.830	.069				
Year 3	44.14 ± 10.55	51.10 ± 10.28	45.38 ± 6.13	2.851	.068				
Year 4	44.71 ± 11.46	53.20 ± 11.04	45.38 ± 3.30	4.475	.017*	.19	.03*	.98	.04*
Year 5	45.43 ± 8.57	53.80 ± 10.24	45.38 ± 3.30	6.489	.003**	.28	.01*	.99	.01*
Year 6	40.71 ± 10.04	52.40 ± 10.22	46.50 ± 5.16	1.676	.198				
**Meaningfulness**									
Year 0	36.86 ± 9.99	43.10 ± 5.06	35.13 ± 9.42	4.821	.012*	.21	.08	.83	.01*
Year 1	38.14 ± 6.16	43.00 ± 5.37	33.25 ± 8.97	8.860	.001**	.38	.12	.14	.00**
Year 2	38.71 ± 4.53	41.10 ± 5.96	33.38 ± 12.20	4.011	.025*	.17	.68	.19	.02*
Year 3	38.86 ± 4.20	39.50 ± 8.64	33.38 ± 10.78	2.613	.084				
Year 4	37.57 ± 5.76	39.70 ± 8.04	33.63 ± 11.03	2.235	.118				
Year 5	38.57 ± 4.80	41.80 ± 6.17	34.00 ± 11.20	4.383	.018*	.19	.47	.26	.01*
Year 6	40.71 ± 5.28	39.70 ± 9.13	34.50 ± 11.70	2.067	.138				

*p < .05; **p < .01; ***p < .001.

## Discussion

Based on the data presented here, we would argue that especially one style of success can be predicted on the basis of earlier dynamics in the sense of coherence: the “Clever” students, who obtain the highest quality of life and seem to be doing the best job of managing their life. They earn the highest income, and are the least overwhelmed by work-related stress. However, they are characterized by a low level of professional competence, and they are also the least satisfied with their chosen career. In order to understand the sources of the differences between the “Clever” and the other styles, it may be useful to analyze the differences between them in the level of the sense of coherence at admission and during their medical studies. The “Clever” are medical students who, compared to the other styles, achieve the highest level of a general sense of coherence throughout their medical education. Unfortunately, we have to admit that a general sense of coherence is not a universal significant predictor of success. However, the students belonging to the “Clever” group have the highest level of comprehensibility and manageability during medical school. What is more, they achieve a higher level of meaningfulness than do the “Bright” students. Fortunately, the three parameters of the sense of coherence (comprehensibility, manageability and meaningfulness) are significant predictors of styles of success over the long term.

Our work suggests that the “Clever” are those medical students who believe that things happen in an orderly and predictable fashion, and they believe that they have the skills needed to take care of things, and that things are manageable. Moreover, the “Clever” believe that life is a source of satisfaction, and that there is good reason to care about what happens. As we know, a sense of coherence correlates with psychological well-being and adaptive coping strategies [23,24,27]. The sense of coherence is also connected with mental health among medical students [34]; its absence is an important risk factor for chronic work-related stress in young physicians, and it is a protective factor for health and correlates with life satisfaction in physicians [38,40]. Our work confirms these results. Antonovsky’s SOC-29 questionnaire can be a tool to identify a specific tendency in career development in terms of: professional competence (State Examination for Medical Doctors), burnout, satisfaction with medicine as a career and quality of life. Based on the SOC questionnaire, we can still predict the course of the medical career even at the moment of admission to medical school. However, the predictions involves belonging to a specific style of success, but not the level of success itself. It would appear that in Polish medical education there are some crucial moments to identify the quality of job adaptation and future career: admission, and the second, fifth and sixth years of medical school. This means that these three styles are shaped at the end of high school education, the end of the theoretical part of medical study, and finally at the end of the whole process of medical education.

It would seem useful to mention here that the SOC level seems to be a useful tool for the early identification of the capability of choosing a specialization [40]. According to our research, this could be a part of job counselling for medical students starting at admission.

According to the literature, the sense of coherence conceptually overlaps with the concept of purpose of life, and is associated with the process of adaptation to negative life events [45]. However, successful adaptation and swift recovery after experiencing adverse life events is related to secure attachment, experiencing positive emotions, and a purpose in life as a third variable [46]. The SOC results measure one aspect of resilience. Our suggestion for future research is to take under consideration these additional two dimensions of resilience, so that we could identify the dynamic process that enables the individual to adapt successfully to adversities over the course of a medical career.

We are aware that the study presents a range of limitations. The data does not propose integrated explanation of the styles of success for the whole medical doctors population. Another limitation of this study is that it does not pertain to dropouts from the medical career, which is one of the success factors mentioned in the literature. One of the strongest points of this attempt is that all of the parameters we have analyzed refer to the same research group, from the beginning of medical school till the fourth year after graduation.

## Conclusions

The most important of our findings implies that the sense of coherence is a psychological concept whose attendant constructs lead to an understanding of the mechanism of functioning in one specific group of physicians: the “Clever.” The different psychological measures that characterize other groups remains a problem for future research. Future studies on the styles of success in a medical career should be conducted on a larger research population and in a wider cultural context, and should take under consideration persons who abandon their medical career.

## Endnote

^a^The Cantril’s Scale, which has been used by a wide variety of researchers since its initial development by Hadley Cantril, is an example of one type of wellbeing assessment. The Scale is known as The Cantril Self-Anchoring Striving Scale or Cantril’s Ladder. The Cantril’s Scale measures wellbeing closer to the end of the continuum representing judgments of life or life evaluation. The person has to imagine ladder with steps numbered from zero at the bottom to 10 at the top. The top of the ladder represents the best possible result and the bottom of the ladder represents the worst possible result.

- Cantril H. *The pattern of human concerns*. New Brunswick, NJ: Rutgers University Press; 1965.
- Diener E, Kahneman, D, Tov W, Arora R: *Income's Differential Influence on Judgments of Life Versus Affective Wellbeing.* Assessing Wellbeing. Oxford, UK: Springer; 2009.
